# Novel Equine Faecal Egg Diagnostics: Validation of the FECPAK^G2^

**DOI:** 10.3390/ani10081254

**Published:** 2020-07-23

**Authors:** Fiona Tyson, Sarah Dalesman, Peter M. Brophy, Russell M. Morphew

**Affiliations:** Institute of Biological, Environmental and Rural Sciences, Aberystwyth University, Aberystwyth SY23 3DA, UK; sad31@aber.ac.uk (S.D.); pmb@aber.ac.uk (P.M.B.); rom@aber.ac.uk (R.M.M.)

**Keywords:** strongyles, nematode, helminth, FEC, faecal egg counting, diagnostic

## Abstract

**Simple Summary:**

Faecal egg counts (FECs) are the standard method of diagnosing the level of intestinal parasites in horses and other grazing animals. Testing before treatment is an important factor in slowing the appearance of drug resistance in these parasites. The FECPAK^G2^, optimised for livestock, allows owners to perform FECs by tapping into remote expertise. However, the performance of the FECPAK^G2^ has yet to be assessed for equids. Therefore, a comparison of the FECPAK^G2^(G2) method with an accepted non-remote equine FEC method (FECPAK^G1^) was performed, using samples of faeces from horses in Wales and New Zealand. The FECPAK^G2^ performed equally as well as the control method (FECPAK^G1^), and this was true regardless of the data’s country of origin. The mean percentage accuracy of the G2 test compared to the control values was 101%. The relative accuracy of the G2 method compared to the control method was not affected by the level of infection and it was concluded that the FECPAK^G2^ method is suitable for performing FECs in horses. It is anticipated that the user-friendliness of the method will increase the uptake of FECs amongst horse owners by the direct use of the technology or through their veterinary practice, likely slowing the development of anthelmintic resistance.

**Abstract:**

Faecal egg counts (FECs) are the standard method of diagnosing the level of parasitic helminth egg shedding in horses and other grazing animals. Testing before treatment is an important factor in slowing the appearance of anthelmintic resistance in nematode parasites. The FECPAK^G2^, optimised for livestock, is reported to allow owners to perform FECs on their own animals without the need for a separate microscope or any specialist knowledge by tapping into remote expertise. However, the performance of the FECPAK^G2^ has yet to be assessed for equids. Therefore, a comparison of the FECPAK^G2^ (G2) method with an accepted equine FEC method (FECPAK^G1^(G1)) was performed, using faecal samples from 57 horses in Wales and 22 horses in New Zealand. There was a significant correlation between the FECs obtained by the two methods (*p* < 0.001) and no effect of the country of origin on the data (*p* = 0.157). The mean percentage accuracy compared to the control values (mean G2 count as a percentage of the mean G1 count, ±SStandard Error (SE)) was 101 ± 4%. There was no significant interaction between the method applied and the country of origin of the data (*p* = 0.814). The relative accuracy of the G2 method compared to the control method (FECPAK^G1^) was not affected by the level of infection (*p* = 0.124) and it was concluded that the FECPAK^G2^ method is a suitable method of performing FECs in horses. It is anticipated that the user-friendliness of the method will increase the uptake of FECs amongst horse owners, either by the direct use of the technology or through their veterinary practice, likely slowing the development of anthelmintic resistance.

## 1. Introduction

It has long been recognised that horses harbour parasites and, since Roman times, these have been identified as a source of disease [1]. Since the 1960s, and with the discovery of additional chemical classes in the 1970s and 1980s, it has been possible to control these parasites using modern anthelmintics [2]. Initially, horse owners were advised to treat all animals routinely every two months with one of these anthelmintics on a schedule designated as interval dosing [3]. Where anthelmintics were available, the interval dosing approach has been successful in effectively controlling clinical diseases caused by *Strongylus vulgaris*, once the most prevalent parasites of horses, in managed equines [1]. However, cyathostomins (small strongyles) filled the niche vacated by the *Strongylus* species to become the primary nematode parasites of horses [4,5], a situation which still prevails [6]. 

Parasitic helminth egg shedding is over-dispersed in horses, with a few individuals shedding the majority of eggs [6,7,8]. Despite this, many owners appear to follow the blanket interval dosing regimen introduced fifty years previously, treating all animals regularly despite unknown infection levels [9]. Such practices are not only wasteful, but also lead to a significant selection pressure for anthelmintic resistance [10].

The control of parasites is an important part of horse husbandry to ensure good health and optimum performance. This is especially pertinent given helminth infections have been demonstrated to be the direct cause of 30% of chronic weight loss syndrome cases in areas of Europe, of which 75% were attributed to larval cyathostominosis [11] which has a mortality rate of up to 50% [12] and has a similar impact on horses across the world [13]. Therefore, new strategies for the sustainable control of equine helminths must be developed to reduce the reliance on anthelmintics, especially with limited prospects of new anthelmintic classes in the near future to target resistant worms.

The resistance of parasitic worms to anthelmintics is an increasing threat to grazing animals worldwide. Within the cyathostomin parasites of horses, a resistance to benzimidazoles or tetrahydropyrimidines has been reported in numerous countries [14] with incidences of multiple anthelmintic resistant helminths in the UK [15]. An important grazing method for delaying the development of anthelmintic resistance is the maintenance of a refugia population of parasites that are not exposed to anthelmintic treatment (for a review of refugia see Sangster, 2001 [16]). Given there is an increasing anthelmintic resistance within the equid sector, and to promote refugia, recommendations have moved away from routine interval dosing towards an integrated parasite management approach, incorporating targeted selective treatment (TST) [1,17], a process heavily reliant on the availability of robust diagnostics, for example, to monitor egg shedding and anthelmintic efficacy via faecal egg counting (FEC).

Various FEC methodologies are currently available, which all involve mixing a specified quantity of faeces with a flotation solution and examining the results using microscopy. Many FEC methods have been widely tested and reviewed for livestock and horses including McMaster [18], Cornell–Wisconsin [19], FLOTAC [20], mini-FLOTAC [21] and FECPAK^G1^ [22]. Unfortunately, to maximise the benefit of these tests, the successful application of any of these methods requires some specialist knowledge in order to identify the eggs from parasitic helminths and this represents a weakness for supporting the sustainable management of parasitic nematodes.

The FECPAK^G1^ method [22] is a commercial faecal egg counting method based on similar principles to the McMaster method [18]. The FECPAK^G1^ differs from the McMaster method by using a larger microscope slide providing an improved lower limit of detection. The FECPAK^G1^ method was initially developed for use in ruminants and as such has been successfully utilised in the ruminant sector since its launch in the early 1990s. The FECPAK^G1^ has since been adapted for equine use from 2004 and is now well utilised in the equid sector [22]. Therefore, the FECPAK^G1^ (G1) was chosen as a comparative FEC for validating future FEC systems not established for equine use.

Recently, the FECPAK^G2^ has been developed, for sheep and cattle, and also used for other ruminants [23], to replace the previously well-established FECPAK^G1^ [24]. FECPAK^G2^ methodology, (outlined in the Methods below) as with alternative methods, floats helminth eggs in a flotation solution prior to microscopy. In addition, the automatic image capturing facility of the FECPAK^G2^ has been utilised in human soil transmitted helminth studies, albeit the preparation method is different [25,26,27]. Importantly, with FECPAK^G2^, the output FEC is imaged by automated microscopy. FEC images are small enough to be captured in a single field of view due to the effect of the meniscus in the imaging cassette, which concentrates helminth eggs in the centre of the FECPAK^G2^ cassette well [28]. The resulting FEC images are uploaded across the internet and analysed remotely, enabling FECs to be performed by farmers themselves, or potentially horse-owners, without specialist knowledge of helminth egg identification. However, the performance of the FECPAK^G2^ has yet to be assessed for equine samples.

Thus, TSTs will focus on only highly infected horses and replace blanket interval dosing and, therefore, minimise the risk of parasitic helminth disease whilst, in turn, reducing the reliance on anthelmintics which should slow the development of anthelmintic resistance [29]. However, to support these new TST practices in the equid sector, improved methods of performing equine FECs are urgently required, especially removing the technical challenges for the novice user, such as overcoming the tendency for lay-users to misidentify helminth eggs [29]. Therefore, the aim of the current work is to validate the use of the FECPAK^G2^ system for equids comparing this to the well-established, previous version, the FECPAK^G1^ [22].

## 2. Materials and Methods

### 2.1. Initial Optimisation

**Figure 1:**
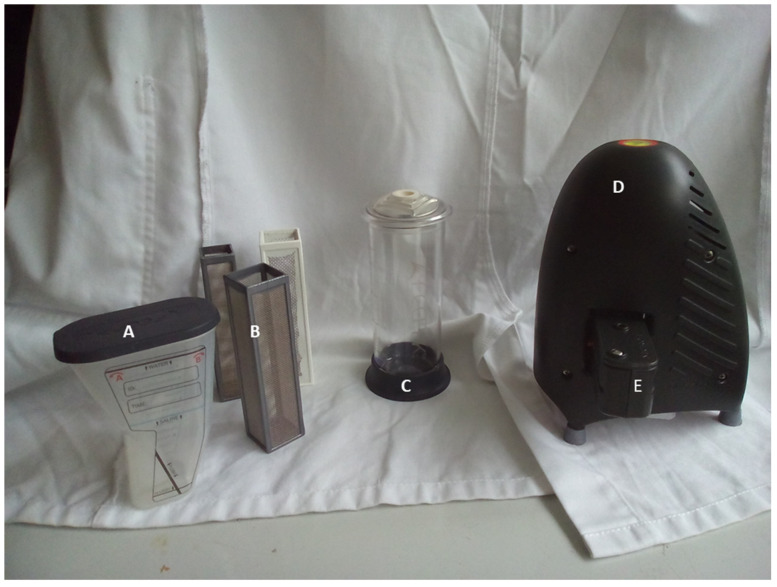
The supplied FECPAK^G2^ equipment. A: Sedimentor; B: 1 mm white pre-filter and 600 and 425 µm silver filters; C: filter cylinder; D: Micro-I imaging device; E: FECPAK^G2^ cassette, inserted into Micro-I.

Figure 1 shows the equipment used for the FECPAK^G2^ system as described below.

Prior to the commencement of the validation, the FECPAK^G2^ (G2) method was optimised for equine faecal samples. The optimum sedimentation time for all nematode eggs to collect in the G2 sedimentors was determined by comparing the FECs from sedimentors allowed to stand for 5, 10, 15, 30, 45, 60, 90, 180 and 1000 min (three sedimentors for each time point). 

The optimum accumulation time (time taken for nematode eggs to float up to the meniscus and congregate in the centre) was determined by preparing G2 samples and programming the Micro-I developer software (Techion Group Ltd., Dunedin, New Zealand) to capture eight images at two minute intervals, imaging immediately after 2, 4, 6, 8, 10, 12 and 14 min. These images were examined to determine when all the eggs had accumulated to the visible area of the well. The second and third cassettes were filled from the same preparation and the imaging procedure repeated. To note, the software settings are not accessible to the end user of the FECPAK^G2^ system, as the Micro-I is programmed to capture images automatically at the correct accumulation interval, and the images are uploaded via the internet for the eggs to be counted by skilled technicians.

### 2.2. FEC Data Generation: Wales, UK

Fresh faecal samples were collected from a total of 57 horses and ponies across mid Wales, UK. A minimum of three faecal balls from a freshly voided faecal pat were collected for each sample, in a plastic bag. Air was excluded from the bag, and the faecal sample was stored at 4 °C before being processed as soon as possible. In all cases, samples were processed within six days. A faecal slurry was created from each well-mixed sample using 50 g faeces and 200 mL water. From each slurry, two FECPAK^G1^ (G1: Techion Group Ltd., Dunedin, New Zealand) preparations were made as follows. Each preparation was generated by suspending 45 mL of faecal slurry in 185 mL of saturated NaCl solution (specific gravity 1.2) and filtering the slurry through a 670 µm mesh, proceeding as in Presland et al. [22]. Each preparation was then used to fill two FECPAK^G1^ slides (1 mL in the counting area, giving a multiplication factor 25), giving four FECs per sample. Helminth eggs were counted using light microscopy at 100× magnification. The arithmetic mean of the four counts formed the control values for each sample. The G1 method provided the control values to validate the G2 method as an alternative to the G1.

Thirty-two of the samples had a FEC of zero and were discarded from future analysis. Four of the samples had a FEC of less than 45 epg and were also discarded as this is below the detection limit for the FECPAK^G2^ (G2) method. A further four samples had a FEC of less than 90 epg, and these were also discarded on the grounds that this would represent only one helminth egg in the G2 protocol, and as two images are produced it was decided that a minimum of two eggs should be expected in order for a valid comparison to be made between the methods and to avoid over-stating the accuracy of the G2 test. This is well below the often-proposed treatment threshold of 200 epg [30]. Therefore, during the validation, equine egg counts ranged from 100 to 3000 epg with the majority of the samples falling between 300 and 1000 epg. A total of 17 slurries with G1 FECs of greater than 90 epg were each used to perform two FECPAK^G2^ (G2: Techion Group Ltd., Dunedin, New Zealand) preparations. Each preparation was generated by spooning faecal slurry into a FECPAK^G2^ sedimentor (Techion Group Ltd., Dunedin, New Zealand) up to the slurry line (12 mL) and filling the sedimentor with water to the fill line (210 mL total) according to the manufacturer’s instructions. This faecal suspension was filtered through a 1 mm filter in a FECPAK^G2^ filter cylinder (Techion Group Ltd., Dunedin, New Zealand) and returned to the sedimentor. The faecal suspension was left to stand for 30 min, as determined during the initial optimisation, and the supernatant was discarded. The sediment was resuspended in 80 mL of saturated NaCl solution and transferred to a FECPAK^G2^ filter cylinder (Techion Group Ltd., Dunedin, New Zealand) fitted with 600 and 425 µm filters (standard FECPAK^G2^ filters per manufacturers’ instructions). An aliquot of 440 µL of the filtered solution was dispensed into each well of two FECPAK^G2^ cassettes (giving a multiplication factor 45) using the FECPAK^G2^ kit supplied pipette (Techion Group Ltd., Dunedin, New Zealand). The cassettes were allowed to stand for six minutes as determined during the initial optimisation. The cassettes were then imaged using the FECPAK^G2^ Micro-I (Techion Ltd., Dunedin, New Zealand). This provided 4 G2 FECs per sample to compare with the 4 FECs from the G1 preparations.

### 2.3. FEC Data Generation: New Zealand

For the New Zealand (NZ) data collection, faecal samples were collected from 22 horses with naturally acquired strongyle infections of at least 90 epg, determined by G1 counts as described above in the UK data collection, with the exception that each preparation was counted only once. From each sample, two G2 preparations were made as described above, with each preparation used to fill one cassette (two G2 FECs per sample).

### 2.4. Statistical Analysis

A statistical analysis was carried out using SPSS version 22.0 (IBM computers Ltd, Armonk, New York State USA.). A Pearson correlation was calculated on the UK data, the NZ data and the combined data to test the correlation between the G1 egg counts and the G2 egg counts. A repeated measures ANOVA (rmANOVA) was used to determine the repeatability of the FEC within each method in the UK and New Zealand samples. As both analyses demonstrated no difference among the repetitions within the sampling methods, the average FEC obtained by each method from each individual was used to compare the G1 and G2 methods. A rmANOVA was then carried out to compare the mean G1 epg with the mean G2 epg (within-subject factor) and the UK and NZ data (between-subject factor) to determine whether the sampling methods differed and if the country in which they were tested affected this outcome. The effect size is reported as partial eta squared (ŋ2*p*). Mauchly’s test of sphericity was used to determine the homogeneity of variance for the rmANOVA, and when heterogeneity was found the more conservative Greenhouse–Geisser correction was used. 

The percentage deviation from the mean G1 count of a sample was calculated separately for each G2 count using the following formula: (G1 mean epg–G2 epg seen)/G1 mean epg × 100. The percentage deviations were converted into positive values, so that an under-reading test would not compensate for an over-reading test. A Pearson correlation was performed on these percentage deviations against FECs from the original G1 egg count to determine if the relative accuracy of the G2 method differs with different infection loads. 

## 3. Results

**Figure 2:**
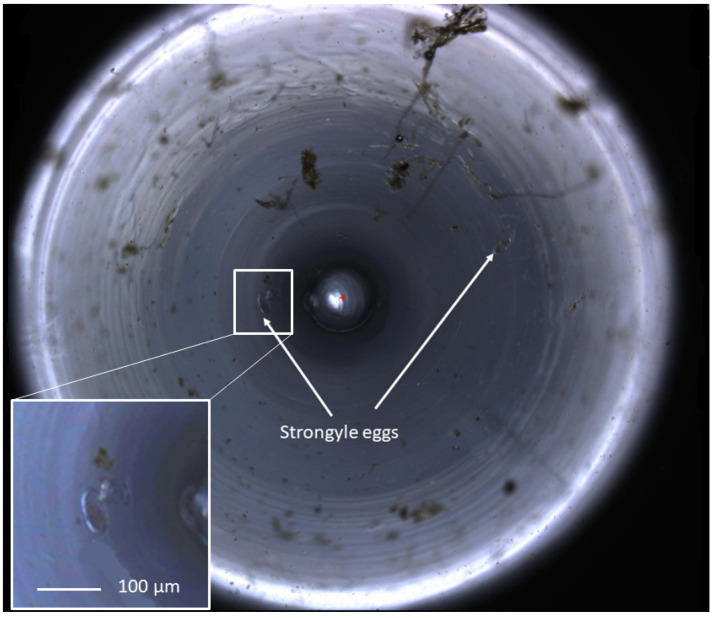
A zoomed-out view of an equine faecal egg count imaged using the FECPAK^G2^ Micro-I. The circular image demonstrates a 3 mm field of view at the top of the meniscus produced in the FECPAK^G2^ Micro-I cassette. Helminth eggs in a flotation solution accumulate to the centre of the well, following the shape of the meniscus. The central circle represents the tip of the light source within the cassette. Equine strongyle eggs are highlighted with white arrows. One of two images produced for each G2 cassette, with each helminth egg seen over the combined two images representing 45 epg, calculated from the dilution factor and the volume of the FECPAK^G2^ cassette. These images are visible only to the technicians who identify the eggs, and they are large file sizes so they can be zoomed in without loss of clarity in order for accurate identification of helminth eggs.

The FECPAK^G2^ Micro-I captures images of the FEC produced using the G2 method and is demonstrated in Figure 2. Both the sedimentation time and accumulation time were optimised for a maximum helminth egg recovery to 30 and 6 min, respectively, as described in Section 2.1 “Initial optimisation” (Data not shown).

A power equation was performed to determine the number of samples needed:

*n* ≥ (((t1 + t2) × √2 × CV)/d)^2^

where *n* = sample size needed

t1 = t value for (*p* < 0.05, error degrees of freedom (d.f.)) = 2

t2 = t value for (*p* = 2 × (1 − *p*), error d.f.) where *p* = the chance of success. We want a 90% chance of success, so *p* = 0.9 and t2 = 1.3

CV = coefficient of variation, and the figure for CV of the G1 counts was used (78%)

d = “treatment success” i.e., if G2 was 95% of G1 that would be considered a success.

The power equation showed that a minimum of 15 samples would be needed, and therefore the 17 UK samples tested would ensure a statistically significant result.

**Figure 3:**
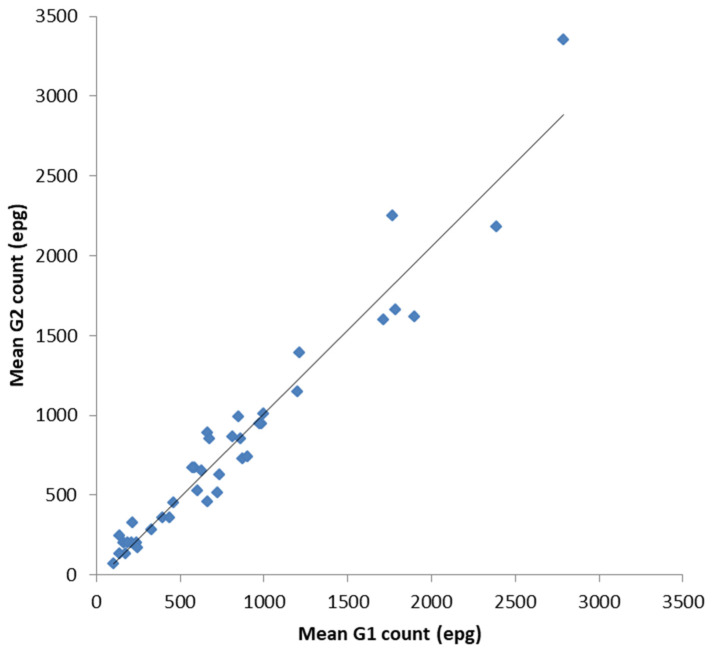
Comparison between the mean G1 and mean G2 counts for each equine faecal sample. The data analysis demonstrates a strong positive correlation between both the established G1 method and the novel G2 method for equid faecal egg counts. The mean percentage accuracy (mean G2 count as a percentage of the mean G1 count, ±SE) was determined at 101 ± 4%.

In order to validate the FECPAK^G2^ (G2) for equids against the FECPAK^G1^ (G1) all data (UK and New Zealand) were combined for a more complete analysis. Following a statistical analysis, there was a significant positive correlation between the mean G1 epg counts and the mean G2 epg counts for each sample of the combined dataset (Figure 3, *r* = 0.971 (CI:0.956, 0.987), *n* = 39, *p* < 0.001). When data were analysed independently, the UK and New Zealand, the same statistically significant correlation was also apparent: the UK samples (r = 0.963 (CI:0.907, 0.990), *n* = 17, *p* < 0.001) and NZ samples (*r* = 0.974 (CI:0.962, 0.993), *n* = 22, *p* < 0.001). The overall similarity between equine FECs on each sample was also calculated. The mean percentage accuracy compared to the control values (mean G2 count as a percentage of mean G1 count, ± SE) was 101 ± 4%.

It was important to determine whether equid FECs performed using the G2 were as consistent as those performed using the G1 control method. Following analysis, there was no significant difference between the repeated samples using either the G1 or the G2 method, including where data for the UK or NZ were analysed independently (Table 1). Repeatability was therefore similar with both methods.

It was also important that the FEC for each sample was comparable between the two methods, G1 and G2. Post statistical analyses revealed there was no significant effect of the method used on the mean epg per equid sample (rmANOVA: F1,37 = 0.052, *p* = 0.821, ŋ2*p* = 0.001). The data from NZ and the UK were also compared independently. The epg was also not affected by the country of origin of the data (rmANOVA: F1,37 = 2.084, *p* = 0.157, ŋ2*p* = 0.053). There was no significant interaction between the method used and the country of origin of the data (rmANOVA: F1,37 = 0.056 *p* = 0.814, ŋ2*p* = 0.002). Such an analysis demonstrates the consistency between the G1 and G2 methods which was independent of the country in which they were performed.

Accuracy of the mean G2 count compared to the control values was not significantly affected by FEC level (*r* = −0.251 (CI: 0.030, −0.472), *p* = 0.124, *n* = 39). Therefore, a lack of significant correlation between the relative accuracy and G1 FEC indicates that the relative accuracy of the G2 method is not dependent on the level of egg shedding.

## 4. Discussion

Faecal samples from 79 horses from the UK and New Zealand were collected in order to determine if the FECPAK^G2^ would be as accurate at monitoring parasitic egg shedding in equids as the widely utilised G1 method. After discarding those samples with zero or very low FECs, samples from 39 horses from both the UK and New Zealand were processed using each of the two methods. The number of samples used in this study was well in excess of the power equation, which required 15 samples to have enough power to detect a difference (if one exists).

Repeat counts on each sample were performed with each method, to determine the repeatability of the G2 method and how this compared to the G1 method. The two methods were then compared for each sample, to assess the relative accuracy of the G2 method over the range of infection levels. If the variability and egg counts were similar between the two methods, the G2 would represent a significant improvement over the previous slide-based G1 version given that no knowledge is required relating to the identification of helminth eggs.

The mean value (±SE) of the G2 counts was 101 ± 4% of the G1 counts. The egg count per sample was equally consistent between the G1 and G2 methods whether looking at the UK data, the NZ data, or the combined dataset. This illustrates that the G2 method performed consistently regardless of the location of sampling or the operative completing the test. Importantly, the accuracy of the G2 method, when compared to the G1, did not significantly change with rising FEC levels across a range of FECs of 100 epg to over 3000 epg. In addition, there was no significant difference between the egg count repeat samples using either method, providing confidence that both methods were reliable and that they were consistently applied to the faecal samples during the validation. The lack of variability among samples in each of the methods also illustrates the validity of using the means of repeated G1 and G2 counts to calculate the percentage accuracy.

In addition to utilising the FECPAK^G2^ system as described in the current research, the potential to use the Micro-I imaging device, alone, to image faecal preparations processed in alternative ways, such as in human studies [25,26,27], offers the future possibility for more sensitive tests with a lower detection limit. 

## 5. Conclusions

In conclusion, the FECPAK^G2^ system represents an improved alternative to conventional slide-based faecal egg counting for horses, producing comparable results to the traditional flotation method of the FECPAK^G1^ counterpart. As the FECPAK^G2^ methodology does not require off-site processing, it represents a more convenient method of performing FECs than other methods currently available and reduces the inaccuracies caused by the problematic storage of samples prior to testing [31]. It is hoped that the availability of such a method will further encourage horse owners to perform FECs for targeted treatment [1] and that this will promote the longevity and sustainability of anthelmintics for controlling the nematode parasites of horses.

## Figures and Tables

**Table 1:** Repeatability of methods of equine faecal egg counting using the FECPAK^G1^ and FECPAK^G2^. A repeated measures ANOVA (rmANOVA) was used to test the repeatability of the G1 and G2 methods on the faecal egg count (FEC) replicates of individual equine samples. The analysis demonstrates no differences between the repeated samples meaning that average FEC for each individual could be used to compare the G1 and G2 methods.

Method and Country	Degrees of Freedom	*F*	*p*
G1 UK	1.9, 30.2	0.785	0.458
G2 UK	3, 48	0.743	0.532
G1 NZ	1, 21	2.253	0.148
G2 NZ	1, 21	1.115	0.303
